# Supplementary material for: Comparison of the MultiViewScope Stylet Scope and the direct laryngoscope with the Miller blade for the intubation in normal and difficult pediatric airways: A randomized, crossover, manikin study
Source: PLoS One. 2020 Aug 13;15(8):e0237593. doi: 10.1371/journal.pone.0237593 (PMC7425958; doi:10.1371/journal.pone.0237593)
Supplement: S3 Fig — (PDF) [file pone.0237593.s003.pdf]

S3 Fig. Subject profile plots: Expert anesthesiologists with difficult pediatric airway

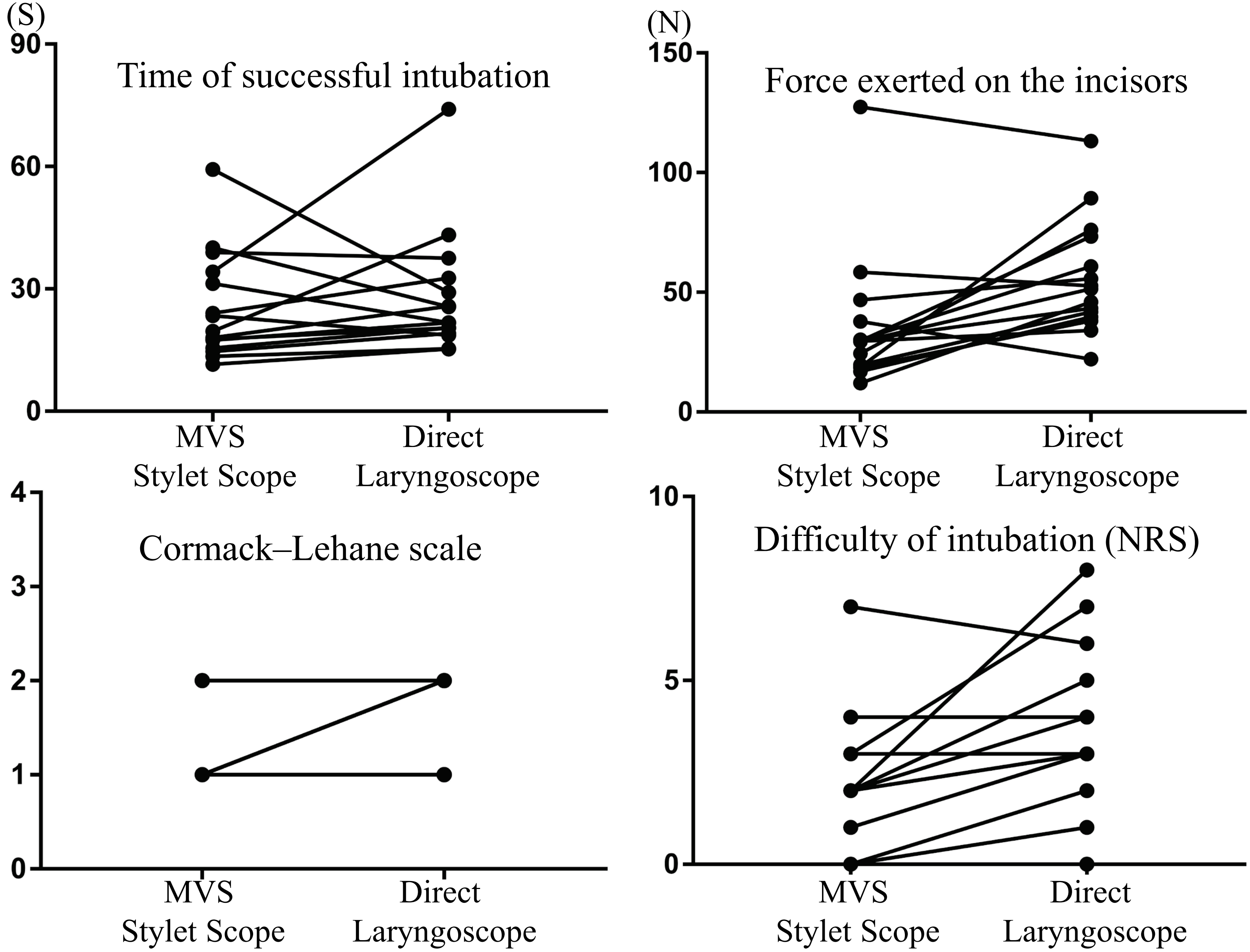

Abbreviations: MVS, MultiViewScope; NRS, numerical rating scale
