# Supplementary material for: Comparison of the MultiViewScope Stylet Scope and the direct laryngoscope with the Miller blade for the intubation in normal and difficult pediatric airways: A randomized, crossover, manikin study
Source: PLoS One. 2020 Aug 13;15(8):e0237593. doi: 10.1371/journal.pone.0237593 (PMC7425958; doi:10.1371/journal.pone.0237593)
Supplement: S1 Table — (PDF) [file pone.0237593.s006.pdf]

S1 Table. Detailed data of the results of expert anesthesiologists with normal pediatric airway.

	Study period		
**Study sequence**	**1**	**2**	**Within-individual difference: SS - DL**
**SS then DL**			
Time (sec), mean (SD)	20.2 (7.2)	24.6 (5.0)	-4.4 (5.0)
Time (sec), *n*	8	8	8
Force (N), mean (SD)	25.8 (8.2)	68.5 (24.9)	-42.7 (28.7)
Force (N), *n*	8	8	8
Cormack–Lehane scale (grade), median (IQR)	1 (1 to 1)	1 (1 to 1)	0 (0 to 0)
Cormack–Lehane scale (grade), *n*	8	8	8
Difficulty of intubation (NRS), median (IQR)	3 (1 to 3.75)	2.5 (0.5 to 3.75)	-0.5 (-1 to 2)
Difficulty of intubation (NRS), *n*	8	8	8
**DL then SS**			
Time (sec), mean (SD)	23.9 (10.4)	25.3 (10.3)	1.3 (11.4)
Time (sec), n	7	7	7
Force (N), mean (SD)	68.9 (12.7)	41.9 (21.7)	-26.9 (21.9)
Force (N), n	7	7	7
Cormack–Lehane scale (grade), median (IQR)	1 (1 to 1)	1 (1 to 1)	0 (0 to 0)
Cormack–Lehane scale (grade), n	7	7	7
Difficulty of intubation (NRS), median (IQR)	2 (0 to 5)	2 (0 to 3)	0 (-3 to 0)
Difficulty of intubation (NRS), n	7	7	7
**Treatment effect**			
Time (sec), mean (95%CI)	-	-	1.5 (-3.2 to 6.3)
Paired analysis	-	-	$P = 0.50$
Force (N), mean (95%CI)	-	-	34.8 (20.4 to 49.2)
Paired analysis	-	-	$P < 0.001$
Cormack–Lehane scale (grade), mean (95%CI)	-	-	0.13 (-0.07 to 0.33)
Paired analysis	-	-	$P = 0.18$
Difficulty of intubation (NRS), mean (95%CI)	-	-	0.38 (-0.78 to 1.53)
Paired analysis	-	-	$P = 0.50$
**Carryover effect**			
Time (sec), mean (95%CI)	-	-	4.4 (-8.7 to 17.6)
Paired analysis	-	-	$P = 0.56$
Force (N), mean (95%CI)	-	-	16.4 (-7.1 to 39.9)
Force (N), n	-	-	$P = 0.24$
Cormack–Lehane scale (grade), mean (95%CI)	-	-	0.02 (-0.32 to 0.35)

Cormack–Lehane scale (grade), n	-	-	*P* = 0.93
Difficulty of intubation (NRS), mean (95%CI)	-	-	-0.04 (-3.47 to 3.40)
Difficulty of intubation (NRS), n	-	-	*P* = 0.99
**Period effect**			
Time (sec), mean (95%CI)	-	-	2.9 (-1.9 to 7.6)
Paired analysis	-	-	*P* = 0.22
Force (N), mean (95%CI)	-	-	7.9 (-6.5 to 22.3)
Paired analysis	-	-	*P* = 0.26
Cormack–Lehane scale (grade), mean (95%CI)	-	-	-0.01 (-0.21 to 0.20)
Paired analysis	-	-	*P* = 0.93
Difficulty of intubation (NRS), mean (95%CI)	-	-	-0.63 (-1.78 to 0.53)
Paired analysis	-	-	*P* = 0.26

Abbreviations: CI, confidence interval; DL, direct laryngoscope; IQR, interquartile range; LSmean, least square mean; NRS, numerical rating scale; SD, standard deviation; SS, MultiViewScope Stylet Scope. *P* values were calculated using ANOVA for crossover design.
