# Supplementary material for: Comparison of the MultiViewScope Stylet Scope and the direct laryngoscope with the Miller blade for the intubation in normal and difficult pediatric airways: A randomized, crossover, manikin study
Source: PLoS One. 2020 Aug 13;15(8):e0237593. doi: 10.1371/journal.pone.0237593 (PMC7425958; doi:10.1371/journal.pone.0237593)
Supplement: S2 Table — (PDF) [file pone.0237593.s007.pdf]

S2 Table. Detailed data of the results of expert anesthesiologists with difficult pediatric airway.

	**Study period**		
**Study sequence**	**1**	**2**	**Within-individual difference: SS - DL**
**SS then DL**			
Time (sec), mean (SD)	24.7 (10.9)	24.9 (7.2)	-0.3 (8.3)
Time (sec), *n*	8	8	8
Force (N), mean (SD)	30.3 (15.6)	48.5 (8.0)	-18.2 (12.7)
Force (N), *n*	8	8	8
Cormack–Lehane scale (grade), median (IQR)	1 (1 to 1)	2 (2 to 2)	-1 (-1 to -1)
Cormack–Lehane scale (grade), *n*	8	8	8
Difficulty of intubation (NRS), median (IQR)	2 (1.25 to 3)	3.5 (3 to 4.75)	-2 (-2.75 to -0.25)
Difficulty of intubation (NRS), *n*	8	8	8
**DL then SS**			
Time (sec), mean (SD)	31.6 (20.9)	26.0 (16.3)	-5.6 (22.0)
Time (sec), n	7	7	7
Force (N), mean (SD)	64.3 (32.8)	41.1 (38.6)	-23.2 (33.5)
Force (N), n	7	7	7
Cormack–Lehane scale (grade), median (IQR)	1 (1 to 2)	1 (1 to 1)	0 (0 to 0)
Cormack–Lehane scale (grade), n	7	7	7
Difficulty of intubation (NRS), median (IQR)	3 (1 to 6)	2 (0 to 2)	-1 (-2 to 0)
Difficulty of intubation (NRS), n	7	7	7
**Treatment effect**			
Time (sec), mean (95%CI)	-	-	2.9 (-6.1 to 12.0)
Paired analysis	-	-	*P* = 0.49
Force (N), mean (95%CI)	-	-	20.7 (6.9 to 34.4)
Paired analysis	-	-	*P* = 0.006
Cormack–Lehane scale (grade), mean (95%CI)	-	-	0.58 (0.35 to 0.82)
Paired analysis	-	-	*P* < 0.001
Difficulty of intubation (NRS), mean (95%CI)	-	-	1.59 (0.57 to 2.60)
Paired analysis	-	-	*P* = 0.005
**Carryover effect**			
Time (sec), mean (95%CI)	-	-	8.0 (-13.9 to 30.0)
Paired analysis	-	-	*P* = 0.53
Force (N), mean (95%CI)	-	-	26.5 (-15.4 to 68.5)
Force (N), n	-	-	*P* = 0.28
Cormack–Lehane scale (grade), mean (95%CI)	-	-	-0.30 (-0.85 to 0.24)

Cormack–Lehane scale (grade), n	-	-	*P* = 0.34
Difficulty of intubation (NRS), mean (95%CI)	-	-	-0.57 (-3.90 to 2.76)
Difficulty of intubation (NRS), n	-	-	*P* = 0.77
**Period effect**			
Time (sec), mean (95%CI)	-	-	-2.7 (-11.7 to 6.4)
Paired analysis	-	-	*P* = 0.53
Force (N), mean (95%CI)	-	-	-2.5 (-16.3 to 11.2)
Paired analysis	-	-	*P* = 0.70
Cormack–Lehane scale (grade), mean (95%CI)	-	-	0.29 (0.06 to 0.53)
Paired analysis	-	-	*P* = 0.02
Difficulty of intubation (NRS), mean (95%CI)	-	-	0.16 (-0.86 to 1.18)
Paired analysis	-	-	*P* = 0.74

Abbreviations: CI, confidence interval; DL, direct laryngoscope; IQR, interquartile range; LSmean, least square mean; NRS, numerical rating scale; SD, standard deviation; SS, MultiViewScope Stylet Scope. *P* values were calculated using ANOVA for crossover design.
