# Supplementary material for: Comparison of the MultiViewScope Stylet Scope and the direct laryngoscope with the Miller blade for the intubation in normal and difficult pediatric airways: A randomized, crossover, manikin study
Source: PLoS One. 2020 Aug 13;15(8):e0237593. doi: 10.1371/journal.pone.0237593 (PMC7425958; doi:10.1371/journal.pone.0237593)
Supplement: S3 Table — (PDF) [file pone.0237593.s008.pdf]

S3 Table. Detailed data of the results of anesthesiology residents with normal pediatric airway.

	Study period		
**Study sequence**	**1**	**2**	**Within-individual difference: SS - DL**
**SS then DL**			
Time (sec), mean (SD)	28.0 (10.1)	28.1 (9.8)	-0.1 (8.8)
Time (sec), *n*	8	8	8
Force (N), mean (SD)	17.6 (6.8)	37.4 (11.4)	-19.9 (11.9)
Force (N), *n*	8	8	8
Cormack–Lehane scale (grade), median (IQR)	1 (1 to 1)	1 (1 to 1)	0 (0 to 0)
Cormack–Lehane scale (grade), *n*	8	8	8
Difficulty of intubation (NRS), median (IQR)	1.5 (0.25 to 2.75)	2.5 (0.25 to 4.75)	- (-2.75 to 0)
Difficulty of intubation (NRS), *n*	8	8	8
**DL then SS**			
Time (sec), mean (SD)	33.8 (11.7)	28.2 (6.2)	-5.6 (8.9)
Time (sec), n	7	7	7
Force (N), mean (SD)	72.8 (38.6)	44.4 (23.4)	-28.4 (40.4)
Force (N), n	7	7	7
Cormack–Lehane scale (grade), median (IQR)	1 (1 to 2)	1 (1 to 1)	0 (-1 to 0)
Cormack–Lehane scale (grade), n	7	7	7
Difficulty of intubation (NRS), median (IQR)	3 (1 to 6)	2 (1 to 3)	0 (-2 to 2)
Difficulty of intubation (NRS), n	7	7	7
**Treatment effect**			
Time (sec), mean (95%CI)	-	-	2.8 (-2.1 to 7.8)
Paired analysis	-	-	$P = 0.24$[a]
Force (N), mean (95%CI)	-	-	24.1 (8.1 to 40.2)
Paired analysis	-	-	$P = 0.006$[a]
Cormack–Lehane scale (grade), mean (95%CI)	-	-	0.20 (-0.03 to 0.44)
Paired analysis	-	-	$P = 0.08$[a]
Difficulty of intubation (NRS), mean (95%CI)	-	-	1.13 (-0.30 to 2.57)
Paired analysis	-	-	$P = 0.11$[a]
**Carryover effect**			
Time (sec), mean (95%CI)	-	-	6.0 (-9.8 to 21.8)
Paired analysis	-	-	$P = 0.51$[a]
Force (N), mean (95%CI)	-	-	62.3 (29.9 to 94.6)
Force (N), n	-	-	$P = 0.005$[a]

Cormack–Lehane scale (grade), mean (95%CI)	-	-	0.16 (-0.23 to 0.55)
Cormack–Lehane scale (grade), n	-	-	P = 0.47[a]
Difficulty of intubation (NRS), mean (95%CI)	-	-	2.16 (-1.29 to 5.61)
Difficulty of intubation (NRS), n	-	-	P = 0.29[a]
**Period effect**			
Time (sec), mean (95%CI)	-	-	-2.8 (-7.7 to 2.2)
Paired analysis	-	-	P = 0.25[a]
Force (N), mean (95%CI)	-	-	-8.5 (-40.7 to 23.6)
Paired analysis	-	-	P = 0.58[a]
Cormack–Lehane scale (grade), mean (95%CI)	-	-	-0.01 (-1.45 to 1.43)
Paired analysis	-	-	P = 0.99[a]
Difficulty of intubation (NRS), mean (95%CI)	-	-	-0.01 (-1.44 to 1.43)
Paired analysis	-	-	P = 0.99[a]
**Comparison of data from study period 1**	**SS**	**DL**	**Mann-Whitney test**
Force (N), median (IQR)	17.6 (12.1 to 21	83.8 (28.1 to 106.	P = 0.006[b]
Force (N), n	8	7	

Abbreviations: CI, confidence interval; DL, direct laryngoscope; IQR, interquartile range; LSmean, least square mean; NRS, numerical rating scale; SD, standard deviation; SS, MultiViewScope Stylet Scope. [a]P values were calculated using ANOVA for crossover design, and [b]P value was calculated using the Mann-Whitney test.
