# Supplementary material for: Comparison of the MultiViewScope Stylet Scope and the direct laryngoscope with the Miller blade for the intubation in normal and difficult pediatric airways: A randomized, crossover, manikin study
Source: PLoS One. 2020 Aug 13;15(8):e0237593. doi: 10.1371/journal.pone.0237593 (PMC7425958; doi:10.1371/journal.pone.0237593)
Supplement: S4 Table — (PDF) [file pone.0237593.s009.pdf]

S4 Table. Detailed data of the results of anesthesiology residents with difficult pediatric airway.

	Study period		
**Study sequence**	**1**	**2**	**Within-individual difference: SS - DL**
**SS then DL**			
Time (sec), mean (SD)	31.6 (13.0)	28.0 (9.9)	3.6 (9.8)
Time (sec), *n*	8	8	8
Force (N), mean (SD)	23.6 (12.2)	43.4 (31.3)	-19.8 (24)
Force (N), *n*	8	8	8
Cormack–Lehane scale (grade), median (IQR)	1 (1 to 1)	2 (2 to 2)	0 (-0.75 to 0)
Cormack–Lehane scale (grade), *n*	8	8	8
Difficulty of intubation (NRS), median (IQR)	2 (1.25 to 3)	3.5 (3 to 4.75)	-1.5 (-2.75 to -0.25)
Difficulty of intubation (NRS), *n*	8	8	8
**DL then SS**			
Time (sec), mean (SD)	28.8 (8.8)	27.5 (5.2)	-1.3 (6.2)
Time (sec), n	7	7	7
Force (N), mean (SD)	57.7 (33.8)	33.9 (26.5)	-23.7 (22.5)
Force (N), n	7	7	7
Cormack–Lehane scale (grade), median (IQR)	1 (1 to 2)	1 (1 to 1)	-1 (-1 to 0)
Cormack–Lehane scale (grade), n	7	7	7
Difficulty of intubation (NRS), median (IQR)	3 (1 to 6)	2 (0 to 2)	-2 (-3 to -1)
Difficulty of intubation (NRS), n	7	7	7
**Treatment effect**			
Time (sec), mean (95%CI)	-	-	-1.2 (-5.8 to 3.5)
Paired analysis	-	-	$P = 0.60$
Force (N), mean (95%CI)	-	-	21.8 (8.7 to 34.8)
Paired analysis	-	-	$P = 0.003$
Cormack–Lehane scale (grade), mean (95%CI)	-	-	0.41 (0.13 to 0.69)
Paired analysis	-	-	$P = 0.007$
Difficulty of intubation (NRS), mean (95%CI)	-	-	1.47 (0.47 to 2.47)
Paired analysis	-	-	$P = 0.007$
**Carryover effect**			
Time (sec), mean (95%CI)	-	-	-3.2 (-19.4 to 13.0)
Paired analysis	-	-	$P = 0.73$
Force (N), mean (95%CI)	-	-	24.7 (-20.0 to 69.4)
Force (N), n	-	-	$P = 0.35$
Cormack–Lehane scale (grade), mean (95%CI)	-	-	0.07 (-0.54 to 0.68)

Cormack–Lehane scale (grade), n	-	-	*P* = 0.84
Difficulty of intubation (NRS), mean (95%CI)	-	-	-0.23 (-3.65 to 3.16)
Difficulty of intubation (NRS), n	-	-	*P* = 0.91
**Period effect**			
Time (sec), mean (95%CI)	-	-	-2.5 (-7.1 to 2.2)
Paired analysis	-	-	*P* = 0.28
Force (N), mean (95%CI)	-	-	-2.0 (-15.0 to 11.1)
Paired analysis	-	-	*P* = 0.75
Cormack–Lehane scale (grade), mean (95%CI)	-	-	-0.16 (-0.44 to 0.12)
Paired analysis	-	-	*P* = 0.23
Difficulty of intubation (NRS), mean (95%CI)	-	-	-0.10 (-1.10 to 0.90)
Paired analysis	-	-	*P* = 0.84

Abbreviations: CI, confidence interval; DL, direct laryngoscope; IQR, interquartile range; LSmean, least square mean; NRS, numerical rating scale; SD, standard deviation; SS, MultiViewScope Stylet Scope. *P* values were calculated using ANOVA for crossover design.
